# Atrial Natriuretic Peptides, Right Atrial Infarction and Prognosis of Patients with Myocardial Infarction—A Single-Center Study

**DOI:** 10.3390/biom11121833

**Published:** 2021-12-04

**Authors:** Michal Kacprzak, Magdalena Brzeczek, Marzenna Zielinska

**Affiliations:** Department of Interventional Cardiology, Medical University of Lodz, 92-213 Lodz, Poland; magdalena.brzeczek@gmail.com (M.B.); marzenna.zielinska@umed.lodz.pl (M.Z.)

**Keywords:** myocardial infarction, atrial infarction, N-terminal proatrial natriuretic peptide

## Abstract

Atrial natriuretic peptide (ANP) is secreted in response to the stretching of the atrial wall. Atrial ischemia most likely impairs the ability of atrial myocytes to produce ANP. Atrial infarction (AI) is rarely diagnosed but not infrequently associated with myocardial infarction (MI). The aim of the study was to assess the association between AI and the prognostic value of N-terminal proANP (NT-proANP) in patients with MI treated with primary percutaneous coronary intervention (PCI). We evaluated data of 100 consecutive patients. Plasma levels of NT-proANP were measured by the ELISA method. ECG recordings were interpreted to diagnose AI according to Liu’s criteria. All patients were followed-up prospectively for 12 months for the manifestation of major adverse cardiovascular events (MACE), defined as unplanned coronary revascularization, stroke, reinfarction or all-cause death. AI was diagnosed in 36 patients. 14% of patients developed MACE. AI did not affect the incidence of MACE or any of its components, nor the patients’ prognosis. NT-proANP revealed to be a strong predictor of death but was not associated with other adverse events. Conclusions: AI in patients with MI treated with primary PCI is not connected with their prognosis nor affects the usefulness of NT-proANP in predicting death during the 12-month follow-up.

## 1. Introduction

There are two natriuretic peptides, structurally similar but genetically distinct, that are produced and secreted mainly by the chambers of the heart: atrial natriuretic peptide (ANP) secreted from the atria and B-type natriuretic peptide (BNP) secreted from the ventricles [1,2]. ANP, like all natriuretic peptides, is synthesized as a preprohormone. The first 25 amino acids comprise a signal sequence, and the next following 126 amino acid peptides constitute proANP, which is the major form of ANP stored in secretory atrial granules. When released from these granules, proANP is immediately split by corin and forms a biologically active 28 amino acid peptide–ANP and inactive N-terminal proANP (NT-proANP). After being secreted in response to the stretching of the atrial wall, ANP enters the coronary sinus and is transported via the circulatory system to all organs possessing suitable receptors (kidneys, lungs, adipose tissue, adrenal glands, brain, heart, testes and vascular smooth muscle tissue). The active ANP is decomposed by the action of neutral endopeptidase and by binding to the natriuretic peptide receptors. The approximate ANP plasma half-time is approximately 2–3 min. Released in equimolar amounts NT-proANP, slowly eliminated by kidneys, appears to have more practical diagnostic use [3,4,5].

Atrial infarction (AI) is a rarely diagnosed disease. Usually, AI is a consequence of an atherosclerotic process, accompanying 0.7–42% of ventricular myocardial infarctions. AI more often appears in the right atrium, accounting for 81% to 98% of cases [6,7]. The occurrence of AI may be associated with a higher incidence of supraventricular arrhythmias (atrial fibrillation, wandering pacemaker, atrial tachycardia or atrial premature complexes) than in ventricular infarctions alone (61–74% vs. 8%), signs and symptoms of heart failure due to loss of ‘atrial kick’ or with thromboembolic events [8,9]. 

Although described for the first time almost 100 years ago, there are still no validated criteria for diagnosing AI, the most recognized electrocardiographic criteria are those developed by Liu et al. in 1961, taking into account the deviation of the P-Ta segment as major criteria, and changes in the morphology of the P wave as minor criteria (Table 1) [10].

There are several reports presenting a correlation of the concentration of ANP with atrial dysfunction due to various causes. In the case of chronic heart failure, plasma ANP levels increase with the severity of heart failure [11]. As ANP is known to be synthesized in an energy-dependent pathway in the atrial myocytes, the decreased intracellular energy supply caused by atrial ischemia most likely impairs the ability of atrial myocytes to produce ANP [12]. In patients with acute myocardial infarction, regardless of the infarct-related artery (IRA), a decrease in plasma ANP levels soon after the incident has been reported [13]. In the animal model of myocardial infarction, the secretion of ANP increases in the first half an hour and decreases substantially during the next hours of observation [14]. These results prove that the rapid release of ANP stored in the secretory granules in the atria, caused by stretching the walls of the atria, is followed by a period of time with an inadequate level of the peptide. Insufficient endogenous ANP during acute myocardial (both ventricular and atrial) infarction might promote left ventricular remodeling, hemodynamic deterioration and worsen patients’ prognosis. 

The aim of the study was to assess the association between AI and the prognostic value of NT-proANP in patients with ST-segment elevation myocardial infarction (STEMI) treated with primary percutaneous coronary intervention (PCI).

## 2. Materials and Methods

### 2.1. Study Population

In the prospective study, we enrolled 100 consecutive patients admitted to the Department of Interventional Cardiology with the diagnosis of STEMI between the years 2017–2019. As the artery providing blood supply to the right atrium typically originates from the 1st segment of the right coronary artery (RCA), to assure the affected atrium will be the right one, we restricted the included patients to those with RCA diagnosed as IRA. Patients with an estimated survival time of less than 6 months, persistent atrial fibrillation, Wolff–Parkinson–White syndrome, Lown–Ganong–Levine syndrome, with an implanted electrotherapy device, mitral stenosis, significant mitral regurgitation, significant tricuspid regurgitation or without successful primary PCI meant as lack of TIMI III flow were excluded from the study. Local ethics committee approved the course of the study, and informed consent was given by all its participants.

### 2.2. Study Protocol

Coronary angiography with subsequent primary PCI was performed within 60 min from admission in all patients. All eligible patients received standard drug therapy (aspirin, ticagrelor or clopidogrel, beta-blockers, ACE-inhibitors and statins). All included patients had a successful primary PCI on IRA with drug-eluting stent implantation or balloon angioplasty. Repeated ECG recordings were interpreted in order to reveal the occurrence of Liu’s criteria of AI. Echocardiography was performed in all patients prior to discharge from hospital. We assessed left ventricular function using the ejection fraction (LVEF) by the modified Simpson method. The following data were also collected for evaluation: age, sex, heart rate, systolic blood pressure, signs of heart failure on admission to hospital (according to the Killip classification) and coronary artery disease (CAD) risk factors (hypertension, diabetes, hyperlipidemia, family history of CAD and cigarette smoking).

All patients were followed up prospectively for 12 months for the occurrence of major adverse cardiovascular events (MACE), determined as necessity to perform unplanned coronary revascularization procedure (PCI or CABG), stroke, reinfarction or death from any cause.

### 2.3. NT-proANP Assay

For determination of NT-proANP plasma concentration, blood was drawn into tubes with EDTA on admission to hospital and on the 4th day of hospitalization. Within 30 min from collection, plasma samples were centrifuged, frozen and stored until assay at temperature −62 °C. A NT-proANP assay was performed by sandwich ELISA (enzyme-linked immunosorbent assay) test, using a commercial kit BioVendor Human NT-proANP ELISA (BioVendor, Brno, Czech Republic). Complete blood count, renal and liver function tests, as well as NT-proBNP, were also determined.

### 2.4. Statistical Analysis

We presented categorical variables as frequencies with percentages, while continuous variables as means ± SD or medians with interquartile range. The Shapiro–Wilk test was used to assess the normal distribution of variables. Non-parametric statistics were used for variables with other than normal distribution. Correlations were assessed using the Pearson correlation coefficient or Spearman’s rank correlation coefficient. Student’s *t*-test, Mann–Whitney U test and Kruskal–Wallis analysis of variance were used to compare continuous variables, whereas chi-squared test with Yates’s correction for continuity was used to assess differences between categorical variables. To determine the association between independent variables and a dichotomous dependent variable (occurrence of MACE), stepwise logistic regression was used. To determine the suitability of NT-proANP levels in adverse cardiac events prediction, we used ROC curves. We performed all statistical analyses using STATISTICA 13.0 (StatSoft Inc., 2017; Tulsa, OK, USA). A *p*-value < 0.05 was considered statistically significant.

## 3. Results

Our cohort consisted of 39 women and 61 men aged 65 (60; 72) years. 79% of patients were in Killip class I. Coronary angiography revealed single-vessel disease in 33% of the group. Drug-eluting stents were implanted in 99% of patients. Other clinical features of the group and concomitant therapy are presented in Table 2.

### 3.1. NT-proANP Levels—Relation to Clinical Data

**Figure 1:**
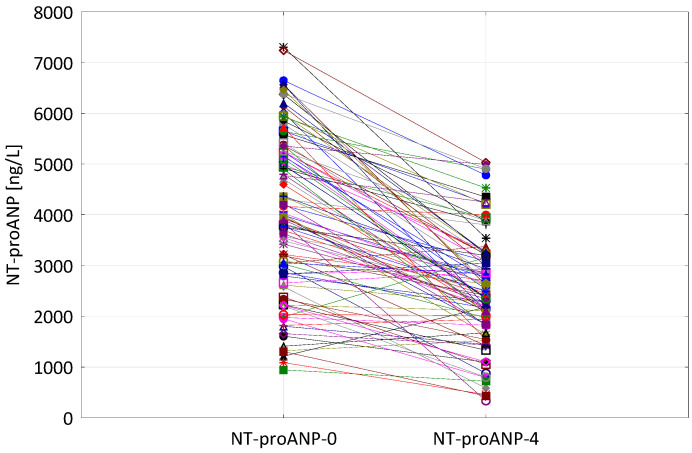
The comparison of NT-proANP concentrations on admission (NT-proANP-0) and on the 4th day of hospitalization (NT-proANP-4) in individual patients.

NT-proANP plasma concentration assessed on admission (NT-proANP-0) was 4039 (2832; 5248) ng/L, while on the 4th day of hospitalization (NT-proANP-4) 2562 (1958; 3199) ng/L. In our group of patients, NT-proANP levels on admission were significantly higher than the values obtained on the 4th day of hospitalization (*p* < 0.0001) (Figure 1).

The occurrence of most CAD risk factors (hypertension, diabetes, smoking status, obesity, family history of cardiovascular diseases), history of previous MI, sex, heart rate, systolic blood pressure and administered therapy did not affect NT-proANP levels (Table 3).

We found moderate, significant correlations between age and NT-proANP levels (r = 0.3770; *p* = 0.0001 and r = 0.48128; *p* < 0.0001, respectively). Patients with diagnosed hypercholesterolemia had lower NT-proANP on the 4th day of hospitalization (*p* = 0.0026), whereas patients in Killip class II–IV on admission had higher NT-proANP concentrations at both time points (*p* = 0.0071 and *p* = 0.0014, respectively). We found no relationship between NT-proANP levels and the extent of atherosclerotic lesions (single- vs. multi-vessel disease). There was a weak, but significant correlation between LVEF and NT-proANP-4 (R = −0.26; *p* = 0.0100).

There were weak to moderate significant correlations between NT-proANP and creatinine (R = 0.279 *p* = 0.0049; R = 0.2003 *p* = 0.0456, respectively), NT-proBNP (R = 0.2136 *p* = 0.0329; R = 0.5473 *p* < 0.0001, respectively) and C-reactive protein (R = 0.2331 *p* = 0.0196; R = 0.2180 *p* = 0.0293, respectively), hemoglobin (R = −0.3337 *p* = 0.0007; R = −0.3319 *p* = 0.0007, respectively) and lymphocyte count (R = −0.2960 *p* = 0.0028; R = −0.3880 *p* = 0.0001, respectively) (Table 4).

### 3.2. Right Atrial Infarction–Relation to Clinical Data

Liu’s electrocardiographic criteria allowed us to diagnose right atrial infarction (RAI) in 36 patients (36%), including 26 men and 10 women. The relationship between the electrocardiographic features of RAI and the presence of risk factors for CAD (family history of cardiovascular diseases, diabetes, arterial hypertension, smoking status and dyslipidemia) has not been established. RAI was also not related to the nutritional status of the patients and their sex. There were no significant relations between the diagnosis of RAI and the segment of the RCA subjected to primary PCI, but the multi-vessel disease was diagnosed significantly more often in this group (*p* = 0.0092). RAI was also not associated with LVEF and the indexed volumes of both left and right atrium.

**Figure 2:**
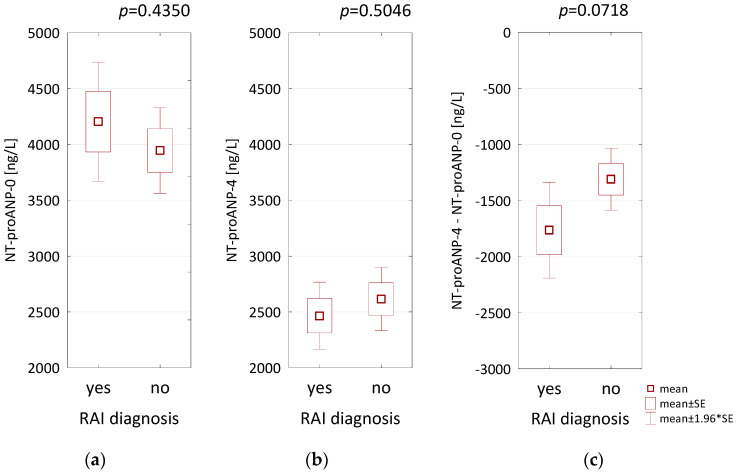
The comparison of NT-proANP concentrations on admission (NT-proANP-0) (**a**), on the 4th day of hospitalization (NT-proANP-4) (**b**) and the difference between two assays (**c**) depending on the diagnosis of right atrial infarction (RAI).

We found that RAI diagnosis was not associated with NT-proANP concentrations, both on admission to hospital and on the 4th day of hospitalization (*p* = 0.4350; *p* = 0.5046, respectively, Figure 2a,b). According to pathophysiological expectations, patients with RAI tended to have a greater decrease in NT-proANP concentrations during the first days of hospitalization, but the difference did not reach predetermined statistical significance (*p* = 0.0718, Figure 2c).

### 3.3. NT-proANP Levels, Right Atrium Infarction—Prognostic Implications

None of the patients died during the index hospitalization. After one year of follow-up, 14 (14%) individuals developed MACE. Six (6%) patients died, there were 5 (5%) cases of reinfarction, 6 (6%) patients underwent unscheduled revascularization procedures.

RAI did not affect the incidence of MACE (*p* = 0.9081) or any of its components (*p* = 0.7655 for unscheduled revascularization, *p* = 0.7743 for reinfarction and *p* = 0.7655 for all-cause death).

**Figure 3:**
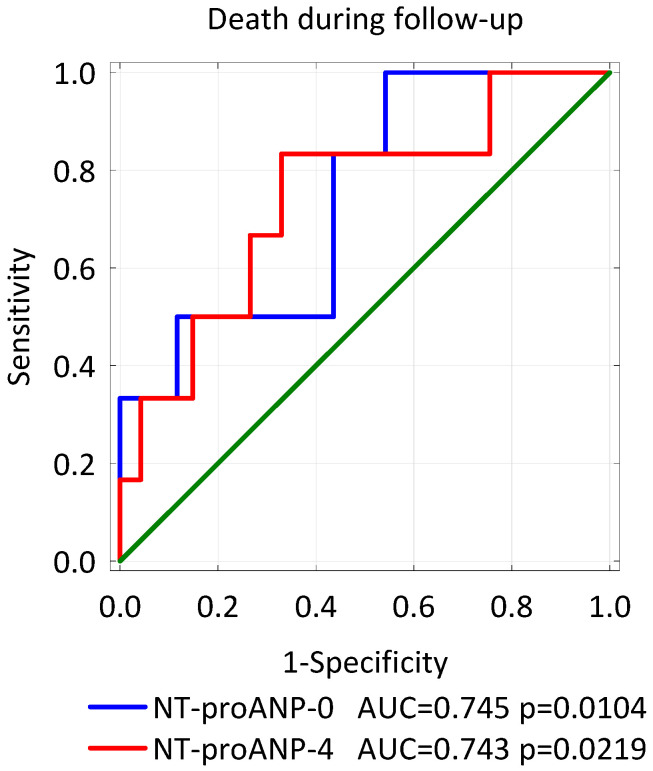
ROC curves-variables tested: the concentration of NT-proANP on admission to the hospital (NT-proANP-0) and on the 4th day of hospitalization (NT-proANP-4) in identifying patients at risk of death during follow-up.

The patients who died during follow-up had higher concentrations of NT-proANP both on admission (*p* = 0.0440) and on the 4th day of STEMI (*p* = 0.0298). Analysis of the ROC curve revealed that both levels of NT-proANP were strong predictors of death after discharge from hospital (AUC = 0.745 *p* = 0.0104 and AUC = 0.743 *p* = 0.0219, respectively, Figure 3).

Patients with reinfarction during follow-up were no different from the others in terms of both NT-proANP levels (*p* = 0.1452; *p* = 0.5198). Analysis of the ROC curves showed that this factor did not differentiate patients at risk of another myocardial infarction (AUC = 0.697 *p* = 0.0567 and AUC = 0.632 *p* = 0.1825, respectively).

Likewise, patients demanding unscheduled revascularization did not differ from the others in terms of NT-proANP concentration (*p* = 0.8262; *p* = 0.5082), which did not allow for the distinction of patients at risk (AUC = 0.537 *p* = 0.8053 and AUC = 0.450 *p* = 0.6407).

**Figure 4:**
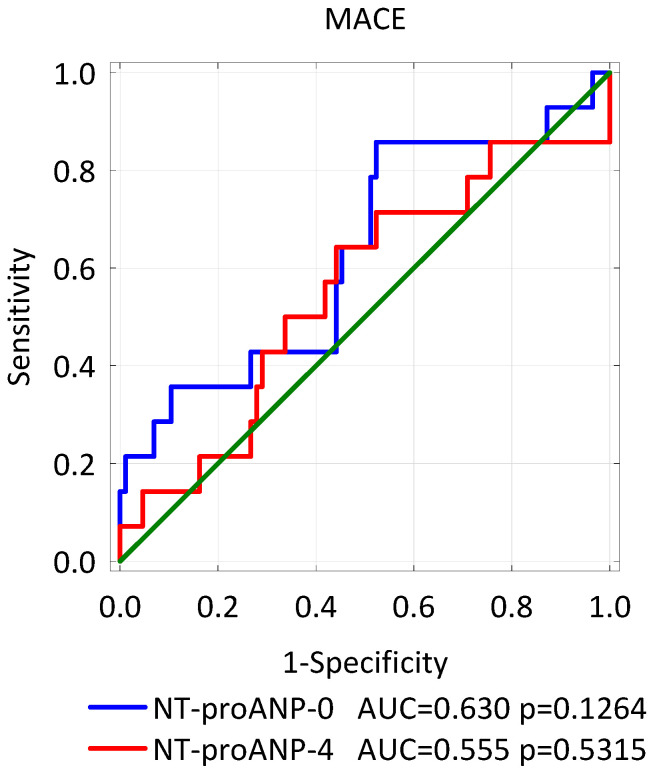
ROC curves–variables tested: the concentration of NT-proANP on admission to the hospital (NT-proANP-0) and on the 4th day of hospitalization (NT-proANP-4) in identifying patients at risk of MACE in the follow-up.

There were no significant differences in the concentration of NT-proANP on admission to the hospital (*p* = 0.12) and on the 4th day of hospitalization (*p* = 0.6538) between patients who developed MACE compared to uneventful survivors. Analysis of the ROC curves showed that this natriuretic peptide did not differentiate patients at risk of all adverse cardiovascular events taken together (AUC = 0.630 *p* = 0.1264 and AUC = 0.555 *p* = 0.5315, respectively, Figure 4).

In multiple logistic regression analysis, among many considered parameters (age, diagnosis of RAI or diabetes, the maximum concentration of troponin T, concentrations of creatinine, NT-proBNP and NT-proANP in both assays and LVEF), none of the factors revealed to be a significant predictor of MACE occurrence.

## 4. Discussion

In our prospective study among patients with STEMI treated with primary PCI of RCA as IRA, we diagnosed RAI in 36% of patients. Although the diagnosis of RAI was associated with multi-vessel coronary artery disease, it had no relation to major coronary risk factors and did not affect prognosis in the one-year follow-up. We also did not notice any significant association with NT-proANP concentration assessed on admission to the hospital and on the 4th day of hospitalization.

In 1994, Yasuda et al. reported a case of a patient with right ventricular infarction with concomitant RAI, which was proven at autopsy. During hospitalization, ANP levels increased gradually but remained within the normal range despite abnormally high right atrial pressure. The authors concluded that atrial infarction probably impairs the ability of atrial myocytes to synthesize ANP, and its insufficient secretion may result in hemodynamic deterioration [12]. To our best knowledge, our study is the first to try to connect the incidence of atrial infarction with ANP secretion in a larger group of patients.

Lu et al. investigated the data of 224 patients to determine the effect of ECG abnormalities suggestive of atrial infarction on mortality after STEMI. They found abnormal P wave morphology (minor Liu criterion) in 35% of the sample population and PR segment displacement in 31%. None of the patients fulfilled any of the major criteria proposed by Liu et al. After adjusting for age, LVEF, peak troponin I and left main disease, PR displacement in any lead was associated with increased 1-year mortality (adjusted OR 6.22 (2.33–18.64)). Observed differences compared to our study may be related to different criteria of atrial ECG abnormalities applied, low participation of Caucasian race patients (22% vs. 99% in our study) or conservative treatment of STEMI in every fourth patient [15].

A different approach was undertaken recently by Yildiz et al., who found atrial infarction in 8.7% of patients with inferior STEMI. The diagnosis was made by atrial branch involvement in coronary angiography, in which RCA was the IRA in 85% of cases. Surprisingly, none of the patients met the major Liu’s criteria. The authors noted that the P-wave duration > 95 ms present in 87% of patients had higher sensitivity and specificity than any other ECG parameters in diagnosing atrial infarction [16].

Laukkanen et al., in a prospective study of 905 middle-aged men without heart failure, proved that NT-proANP could be considered as at least as strong a risk predictor as NT-proBNP in predicting death and the incidence of atrial fibrillation or heart failure [17].

Before the era of widespread invasive treatment of myocardial infarction, Omland et al., in a group of 139 patients treated conservatively (thrombolytic therapy only in 46% of patients), proved that NT-proANP was a significant and better prognostic factor than ANP in predicting death during one year of follow-up after myocardial infarction [18].

Hall et al. proved that 76 patients who died one year after myocardial infarction treated with thrombolysis had higher plasma levels of NT-proANP than those who survived. The increased risk of death associated with higher NT-proANP levels was independent of a history of prior MI, hypertension, diabetes, serum creatinine or atrial fibrillation. Serial measurements of NT-proANP during the first hours after enrollment showed a gradual decrease in its concentration [19].

Similar results were presented by Otterstad et al., who evaluated the impact of NT-proANP and echocardiographic parameters on prognosis after myocardial infarction treated conservatively. A total of 834 patients, of whom 64% received thrombolysis, with LVEF ≥40% were followed up for 24 months. A combined primary end-point defined as cardiac death, recurrent non-fatal myocardial infarction and heart failure requiring hospitalization or treatment with an angiotensin-converting enzyme inhibitor and diuretic therapy was observed in 102 patients. Baseline NT-proANP, but not echocardiographic left ventricular volumes predicted adverse cardiac events [20]. Unfortunately, there are no studies on the prognostic role of NT-proANP in patients after myocardial infarction treated invasively. In our group of well-treated patients, NT-proANP assessed both on admission and on the 4th day of STEMI proved to be a strong predictor of death in one-year follow-up, but it was not associated with other adverse events.

### Limitations of the Study

The most important limitation of the study is the small group of patients; therefore, our results should be interpreted with caution. The diagnosis of atrial infarction was based on the criteria by Liu et al., which have not been validated. The lack of generally accepted and validated criteria for the diagnosis of atrial infarction made the comparison of the obtained results much more difficult. To assure the affected atrium will be the right one, we restricted the included patients to those with RCA diagnosed as IRA. In addition, patients with persistent atrial fibrillation who have a worse prognosis were excluded. 

## 5. Conclusions

Right atrial infarction in patients with STEMI treated with primary PCI of RCA as IRA is not connected with their long-term prognosis, nor affects the prognostic usefulness of NT-proANP in this group of patients.

## Figures and Tables

**Table 1:** Electrocardiographic diagnostic criteria for atrial infarction based on Liu et al. [10].

Major	1. P-Ta-segment elevation > 0.5 mm in leads V5–V6 and reciprocal depression of P-Ta segment in V1 and V2 leads
	2. P-Ta-segment elevation > 0.5 mm in lead I and reciprocal depression of P-Ta segment in II and III leads
	3. P-Ta-segment depression > 1.5 mm in precordial leads and >1.2 mm in leads I, II, III associated with any kind of atrial arrhythmia
Minor	Abnormal P-waves, flattering of P-wave in M-shape or W-shape, irregular or notched P-wave

**Table 2:** Patient characteristics, laboratory test, echocardiography findings and concomitant treatment in the whole study group and by subgroups depending on the diagnosis of right atrial infarction.

Patient Characteristic	N = 100	Right Atrial Infarction (N = 36)	No Right Atrial Infarction (N = 64)	*p*
Age [years]	65 ± 10	64 ± 11	66 ± 10	0.277
Women	39 (39%)	10 (28%)	29 (45%)	0.084
Heart rate [beats/min]	70 (62; 80)	70 (64; 80)	70 (59; 80)	0.437
Systolic blood pressure [mmHg]	140 ± 24	140 ± 29	141 ± 22	0.849
Killip class II–IV	21 (21%)	4 (11%)	17 (27%)	0.069
Hypertension	64 (64%)	20 (56%)	44 (69%)	0.187
Diabetes	28 (28%)	10 (28%)	18 (28%)	0.970
Hyperlipidemia	22 (22%)	5 (14%)	17 (27%)	0.142
Family history of CAD	21 (21%)	8 (22%)	13 (20%)	0.822
Current or former smoker	61 (61%)	24 (67%)	37 (58%)	0.384
Troponin T max [ng/L]	1990 (738; 4198)	2054 (622; 3813)	1968 (770; 4436)	0.906
Serum creatinine [μmol/L]	87 (70; 107)	92 (79; 107)	85 (69; 104)	0.239
NT-proANP-0 [ng/L]	4038 ± 1582	4204 ± 1630	3945 ± 1559	0.435
NT-proANP-4 [ng/L]	2561 ± 1074	2466 ± 920	2616 ± 1156	0.505
NT-proBNP [ng/L]	924 (422; 1782)	985 (260; 1816)	923 (467; 1705)	0.511
Atrial fibrillation during hospitalization	16 (16%)	4 (11%)	12 (19%)	0.317
Multi-vessel disease	**67 (67%)**	**30 (83%)**	**37 (58%)**	**0.009**
EF [%]	51 (47; 56)	50 (47; 55)	53 (46; 57)	0.469
LAVi [mL/m^2^]	37 (31; 44)	38 (31; 44)	37 (30; 44)	0.968
RAVi [mL/m^2^]	25 (19; 31)	27 (20; 31)	23 (19; 30)	0.436
Concomitant therapy:				
Aspirin	99 (99%)	36 (100%)	63 (98%)	0.769
P2Y12 receptor inhibitors	100 (100%)	36 (100%)	64 (100%)	
GP IIb/IIIa blocker	58 (58%)	22 (61%)	36 (56%)	0.636
Statins	98 (98%)	35 (97%)	63 (98%)	0.743
Beta-blockers	91 (91%)	33 (92%)	58 (91%)	0.850
ACE-inhibitors	94 (94%)	35 (97%)	59 (92%)	0.563

ACE = angiotensin-converting enzyme; CAD = coronary artery disease; EF = ejection fraction. GP = glycoprotein; LAVi = left atrial volume index; NT-proANP-0 = N-terminal pro-atrial natriuretic peptide on admission to hospital; NT-proANP-4 = N-terminal pro-atrial natriuretic peptide on the 4th day of hospitalization; NT-proBNP = N-terminal pro-B-type natriuretic peptide; RAVi = right atrial volume index. Statistically significant differences (*p* < 0.05) are shown in bold.

**Table 3:** Association between patient characteristics and NT-proANP concentrations on admission (NT-proANP-0) and on the 4th day of hospitalization (NT-proANP-4).

		NT-proANP-0 [ng/L]		NT-proANP-4 [ng/L]	
Sex	men	3816 (2584; 5245)	*p* = 0.3964	2453 ± 1127	*p* = 0.2089
	women	4165 (3484; 5249)		2731 ± 976	
Nutritional status	normal	4597 (3571; 5357)	*p* = 0.0915	2641 (2112; 3881)	*p* = 0.1817
	over-weight	3694 (2202; 4976)		2413 (1824; 3163)	
	obesity	4492 (2831; 5362)		2400 (1537; 2996)	
Hypertension	yes	4000 (2705; 5203)	*p* = 0.7988	2485 ± 1130	*p* = 0.3428
	no	3755 (2953; 5308)		2699 ± 968	
Diabetes	yes	3765 ± 1462	*p* = 0.2833	2416 ± 1039	*p* = 0.4010
	no	4145 ± 1623		2618 ± 1090	
Hyperlipidemia	yes	3467 ± 1580	*p* = 0.0546	**1961 ± 1095**	***p* = 0.0026**
	no	4200 ± 1555		**2731 ± 1012**	
Family history of CAD	yes	3871 ± 1542	*p* = 0.5875	2292 ± 1041	*p* = 0.1983
	no	4083 ± 1599		2633 ± 1078	
Current or former smoker	yes	3793 ± 1565	*p* = 0.0515	2465 ± 1079	*p* = 0.2644
	no	4423 ± 1551		2713 ± 1064	
Killip class	I	**3754 (2375; 5235)**	***p* = 0.0071**	**2389 ± 993**	***p* = 0.0014**
	II–IV	**4976 (3989; 5646)**		**3214 ± 1143**	
Multi-vessel disease	yes	4073 ± 1562	*p* = 0.7590	2629 ± 1050	*p* = 0.3781
	no	3969 ± 1644		2426 ± 1128	

Statistically significant differences (*p* < 0.05) are shown in bold.

**Table 4:** Association between patient characteristics, laboratory tests, echocardiography findings and NT-proANP concentrations on admission (NT-proANP-0) and on the 4th day of hospitalization (NT-proANP-4).

	NT-proANP-0 [ng/L]		NT-proANP-4 [ng/L]	
	r or R	*p*	r or R	*p*
Age	**r = 0.3770**	**0.0001**	**r = 0.4813**	**<0.0001**
Heart rate [beats/min]	R = −0.029	0.7743	R = −0.0522	0.6060
Systolic blood pressure [mmHg]	r = −0.0696	0.4911	r = −0.1283	0.2034
Serum creatinine [μmol/L]	**R = 0.279**	**0.0049**	**R = 0.2003**	**0.0456**
NT-proBNP [ng/L]	**R = 0.2136**	**0.0329**	**R = 0.5473**	**<0.0001**
C-reactive protein [mg/L]	**R = 0.2331**	**0.0196**	**R = 0.2180**	**0.0293**
Hemoglobin [g/dL]	**R = −0.3337**	**0.0007**	**R = −0.3319**	**0.0007**
Lymphocyte count [10^3^/µL]	**R = −0.2960**	**0.0028**	**R = −0.3880**	**0.0001**
Troponin T max [ng/L]	R = 0.1346	0.1819	**R = 0.2737**	**0.0058**
LVEF [%]	R = −0.1003	0.3208	**R = −0.2564**	**0.0100**

Statistically significant correlations (*p* < 0.05) are shown in bold.

## Data Availability

The data presented in this study are available on request from the corresponding author.
